# Testing Cost-Benefit Models of Parental Care Evolution Using Lizard Populations Differing in the Expression of Maternal Care

**DOI:** 10.1371/journal.pone.0054065

**Published:** 2013-02-07

**Authors:** Wen-San Huang, David A. Pike

**Affiliations:** 1 Department of Zoology, National Museum of Natural Science, Taichung, Taiwan; 2 Department of Life Sciences, National Chung Hsing University, Taichung, Taiwan; 3 School of Marine and Tropical Biology, James Cook University, Townsville, Australia; Institut Pluridisciplinaire Hubert Curien, France

## Abstract

Parents are expected to evolve tactics to care for eggs or offspring when providing such care increases fitness above the costs incurred by this behavior. Costs to the parent include the energetic demands of protecting offspring, delaying future fecundity, and increased risk of predation. We used cost-benefit models to test the ecological conditions favoring the evolution of parental care, using lizard populations that differ in whether or not they express maternal care. We found that predators play an important role in the evolution of maternal care because: (1) evolving maternal care is unlikely when care increases predation pressure on the parents; (2) maternal care cannot evolve under low levels of predation pressure on both parents and offspring; and (3) maternal care evolves only when parents are able to successfully defend offspring from predators without increasing predation risk to themselves. Our studies of one of the only known vertebrate species to exhibit interpopulation differences in the expression of maternal care provide clear support for some of the hypothesized circumstances under which maternal care should evolve (e.g., when nests are in exposed locations, parents are able to defend the eggs from predators, and egg incubation periods are brief), but do not support others (e.g., when nest-sites are scarce, life history strategies are “risky”, reproductive frequency is low, and environmental conditions are harsh). We conclude that multiple pathways can lead to the evolution of parental care from a non-caring state, even in a single population of a widespread species.

## Introduction

Parental care should evolve when the benefits of providing care increase offspring survival above the costs of reduced survival and future reproduction of adults [1]–[3]. Offspring benefit from parental care through an increased chance of survival, which is relatively easy to measure, but the costs for parents are complex, and thus much more difficult to detect [4]. Parental costs are twofold: (1) reproductive costs, including loss of mating opportunities when providing long-term parental care, which can decrease future fecundity or reduce the number of offspring produced during the next reproductive event due to lower energy intake; and (2) survival costs, in which long-term parental care can reduce survival of the parent. Evolutionary theory suggests that parents will adjust their parental care expenditure in relation to the variation in costs to themselves and the benefits to their offspring, so as to maximize fitness [5].

Several mathematical models of parental care have been proposed, most of which involve cost-benefit tradeoffs between the parents and offspring [1]–[2], [5]–[8]. However, one of the principle constraints with predicting the circumstances under which parental care evolves is that the ecological form of the cost and benefit functions is unknown, making models difficult to test empirically [2]. Consequently, models are normally tested using focal species which vary in the type or intensity of care provided (but in which all populations express care to some degree), or to examine interspecific variation in care strategies. Because parental care is either absent altogether or present in the vast majority of species, our capacity to understand the direct ecological influences of parental care evolution at the population level is limited.

Long-tailed skinks (*Eutropis longicaudata*) are widely distributed throughout southeast Asia, but only one insular population is known to display maternal care [9]; this presents a unique opportunity for testing how ecological circumstances can contribute to parental care evolution. On Orchid Island (Taiwan), nesting female long-tailed skinks guard their nests during incubation, whereas females in at least 13 other populations abandon the nest immediately after laying eggs [9]. This pattern is driven by significantly higher levels of predation pressure on Orchid Island, necessitating maternal care to ensure reproductive success [9]. We developed cost-benefit models of parental care, which we tested using ecological data from the population which provides maternal care and two other populations lacking maternal care. We focus on the fitness consequences of maternal care, where mothers benefit by increasing egg hatching rates but incur a cost in terms of the survival of the nest-guarding female and reduced opportunity for future reproduction. This extends other models, in which parental care evolves solely in response to selection on trade-offs between growth rate and future reproduction, or the relationship between parental care and fecundity [2]. Eventually, a balance between the costs and benefits should lead to parental care evolving from a non-caring ancestor, and if the long-term benefits outweigh the costs, then parental care should be maintained. Our study suggests that natural selection acting directly on the female and her ability to produce offspring exerts a strong influence on the evolution of maternal care. We integrate our findings into existing predictive frameworks that propose mechanisms leading to the evolution of parental care [1], and find support for some hypotheses, but not others.

## Methods

### Ethics statement

This study was approved by the Taiwanese National Museum of Natural Science Animal Care and Use Committee (Protocol Permit NMNSHP02-002).

### Study populations and empirical data

From 2001–2010 we collected clutch size data for *Eutropis longicaudata* on Orchid Island, located 60 km southeast of Taiwan (22°02′N, 121°34′E); Green Island, located 33 km southeast of Taiwan (22°40′N, 121°28′E); and mainland Taiwan (Santimen, Pingtung County; 22°42′N, 120°38′E). At each location long-tailed skinks nest within drainage holes running through a concrete retaining wall located along a mountain road. For full details of study sites and methods, see [9]–[11]. Incubating eggs fail due to a variety of reasons, including fungal infections, or predation by ants or egg-eating snakes (*Oligodon formosanus*) [9]. Egg-eating snakes, however, are the major egg predator and the maternal care expressed by Orchid Island long-tailed skinks is aimed at deterring snakes from eating lizard eggs, which are more abundant on this island than our other two study sites [9]. We conducted a manipulative experiment to determine the fitness effects of excluding snake predators (as mother lizards do on Orchid Island) [9]. This entailed gluing mesh over the pipes housing lizard nests; by doing so, we could estimate the fitness effects that maternal care would provide in our two study populations which do not display maternal care (Table 1) [9]. We also conducted these trials on Orchid Island to serve as controls (Table 1) [9].

**Table 1:** Predation, competition, and parental care attributes of three long-tailed skink populations.

Variable	Location		
	Mainland Taiwan	Green Island	Orchid Island
Individual predation	Strong	Weak	Weak
Egg predation	Weak	Weak	Strong
Parental care	No	No	Yes
Clutch size (eggs)	6.7	6.0	6.5
Hatching success with parental care	59%	55%	81%
Hatching success without parental care	62%	51%	18%
Relative change in hatching success with care	−3%	4%	63%
Cost-benefit model without maternal care	1.15	0.06	−1.83
Cost-benefit model evolving maternal care	0.95	0.30	2.27

Individual predation pressure was assessed using the number of lizard predator species present in each location; intensity of egg predation was estimated using numbers of egg-eating snakes (*Oligodon formosanus*) [33] encountered during our study; and intra-specific competition was estimated using skink density. Data for hatching success with and without parental care are from [9].

### Model overview

We used an evolutionary stable strategy (ESS) game theory model [12]. The game theory model predicts that the selective pressures maintaining parental care by both sexes, and desertion by one parent, affects investment by the other parent (Table 2) [12]. We can use the game theory model to define four different evolutionary stable strategies for parental care evolution (ESS): (1) both parents express parental care, and if the costs outweigh the benefits, either parent can abandon care; (2) the female deserts the offspring while the male provides care; (3) the female provides care for the offspring while the male deserts; or (4) both sexes desert the nest resulting in no parental care. Because parental care by male reptiles is virtually nonexistent [1], here we focus on the conditions under which maternal care is likely to evolve (ESS1 and ESS3). The long-tailed skink populations on Green Island and mainland Taiwan are described by evolutionary stable strategy ESS 1: both parents desert the nest, whereas the Orchid Island population is described by ESS 3: the female cares for incubating eggs and the male deserts (Table 2). Because the payoff from these two strategies relates to egg numbers and egg survival, we can calculate the benefits in terms of numbers of eggs produced and the proportion of those eggs that hatch into baby lizards.

**Table 2:** Payoff matrix and four evolutionary stable strategies (ESS) from the game theory model [12].

			Female	
			Care	Desert
Male	Care	♀	wP2	WP1
		♂	wP2	WP1
	Desert	♀	wP1	WP0
		♂	wP1 (1+p)	WP0 (1+p)

W: egg without care; w: egg with care; P0: egg survival without parental care; P1: egg survival with uni-parental care; P2: egg survival with bi-parental care; P2> P1> P0; p: male deserts and then obtains another mating opportunity. ESS1: both sexes show care; wP2> WP1, otherwise female deserts; wP2> wP1 (1+p), otherwise male deserts. ESS2: female deserts and male cares; WP1 > wP2, otherwise female cares; WP1 > WP0 (1+P), otherwise male deserts. ESS3: female cares and male deserts; wP1 > WP0, otherwise female deserts; wP1 (1+p) > wP2 otherwise male cares. ESS4: both sexes desert; WP0 > wP1, otherwise the female cares; WP0 (1+p) > WP1, otherwise the male cares.

### Benefits of parental care

The benefit of parental care is the successful production of offspring. In long-tailed skinks, the proportion of eggs laid that produce hatchlings is a function of incubation temperature [11] and the probability that the eggs will be located and consumed by predators [10]. Eggs exposed to relatively moderate temperatures have high hatching success ratios [11], which increases the benefit of providing parental care. Similarly, parental care maintains a benefit when females successfully attack and deter egg-eating snake predators [9], [10].

### Costs of maternal care

The costs of maternal care are fourfold: (1) the energetic cost of producing a clutch; (2) delay of future mating opportunities (when a female remains with the eggs, she delays opportunities for mating and acquiring the energy necessary for producing her next clutch); (3) anti-predator ability (the ability of a lizard to defend the nest from obligate egg predators); and (4) risk of predation (maternal care could reduce survival through increased predator exposure during care). When considering these costs as point values in a model, the total costs cannot exceed the value of the female herself.

### Model development

The model without maternal care (which is representative of the long-tailed skink populations on mainland Taiwan and Green Island) is:
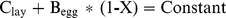
(1)Where C_lay_ is the cost of laying a clutch of eggs by a mother lizard, B_egg_ is the benefit of egg-hatching ratios, and X is the proportion of eggs that fail to hatch.

We assumed that a mother lizard represents ten points, and that laying a clutch of eggs costs the female 30% of her body mass [10], or three points. If all of the eggs in a clutch hatch, the female benefits ten points; this value can be adjusted based on egg hatching rates. We assumed that a female lays eggs twice during her lifetime, and at least one hatchling from each clutch reaches adulthood. This means that in her lifetime each female will replace herself and one mate (20 lifetime points), thereby maintaining a stable population size. These assumptions can be adjusted based on life history attributes; for example, if a female lays four clutches of eggs in her lifetime, then each clutch would represent five points.

The model evolving parental care (which is representative of the long-tailed skink population on Orchid Island) is:
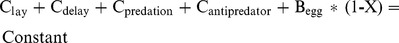
(2)Where C_delay_ is the cost to a female in terms of delaying reproduction while guarding eggs, C_predation_ is the risk of the female provisioning care being preyed upon, and C_antipredator_ is the cost the female incurs when using antipredator behaviors.

If a female develops maternal care, then the costs relative to not providing care include the cost of future reproduction, the risk of predation, and the cost of deterring predators. Delaying future reproduction may be the most important cost for females in our study system [13]. Consequently, C_delay_  = −3 (in Equation 1, we assumed that a clutch represents 3 points, and the greatest benefit to a female would be a clutch that is not preyed upon by snakes, and thus all eggs hatch. In this case X = 0 and −3+6 * (1–0)  = 3. If the entire clutch is consumed by snakes, then X = 1 and −3+6 * (1–1)  = −3; a female loses at most 3 points by delaying the second clutch). C_predation_ and C_antipredator_ are dependent on the female's ability to escape lizard predators and to deter egg predators, respectively. These costs sum to at most 7 points because females invest 3 in the clutch. In our study system, female lizards easily deter egg predators (the reptile egg-eating snake *Oligodon formosanus*, which does not eat lizards; [9], [10]), so we assume C_antipredator_ to be negligible (i.e., 0).

## Results

### Testing the model without parental care

We solved the model without parental care (Equation 1) using empirical data (Table 1). The benefits of not providing care outweigh the costs for the mainland and Green Island populations, but not for the Orchid Island population (Table 1). For the Orchid Island population to persist in the absence of maternal care (i.e., to obtain a positive value for the equation) would require either a decrease in C_lay_ or an increase B_egg_. To obtain a positive value for the equation, X would have to be above 0.46, or, given the current hatching success rate of eggs in the absence of maternal care (18%), females would have to increase their clutch size to >17 eggs (the known maximum clutch size is 13 eggs; [14]). With lower clutch sizes and given the high rate of egg predation in the absence of maternal care, long-tailed skinks would become extirpated from Orchid Island if the eggs were not protected from predation during incubation.

### Testing the model evolving maternal care

We solved the model evolving maternal care (Equation 2) using empirical data (Table 1). Our experimental exclusion of egg predators, which simulates maternal defense of the nest site, revealed that when eggs are protected from predation, B_egg_ increases from 18% to 81% on Orchid Island, but in the other two populations there is negligible change (<4% change; Table 1 [9]). The benefits of providing care thus outweigh the costs only for the Orchid Island population. The absolute value for the Green Island population is higher for the model evolving maternal care than for the model not evolving maternal care (Table 1), but the difference is so small that when using the empirically-derived hatching success data as input (instead of data from the predator exclusion experiment; Table 1), the values for the models with and without maternal care are equal (i.e., 0.06). Lizards on mainland Taiwan and Green Island should therefore continue burying their eggs in the soil and abandoning them after oviposition (as is common throughout most of the species' range).

## Discussion

A major objective in behavioral and evolutionary ecology is to understand how animals make decisions in complex environments, and the strategies adopted by animals under realistic conditions [15]. Our cost-benefit model used an optimization framework [12], [15], [16] to examine the evolution of maternal care in complex environments, and revealed four major insights: (1) maternal care can evolve from a non-parental caring ancestor if the benefits outweigh the costs; (2) in species lacking anti-predator behaviors, the benefits of maternal care can increase with ecological or environmental factors; (3) the benefits and costs of maternal care increase when individuals actively defend their nests against predation; and (4) predators play important roles in the evolution of maternal care. The evolution of parental care represents a considerable evolutionary challenge: to change behavior in a manner that interacts positively with offspring. Nonetheless, phylogenetic studies from a wide range of taxa all suggest that this is the pathway by which parental care evolves (reviewed by [2]).

**Figure 1:**
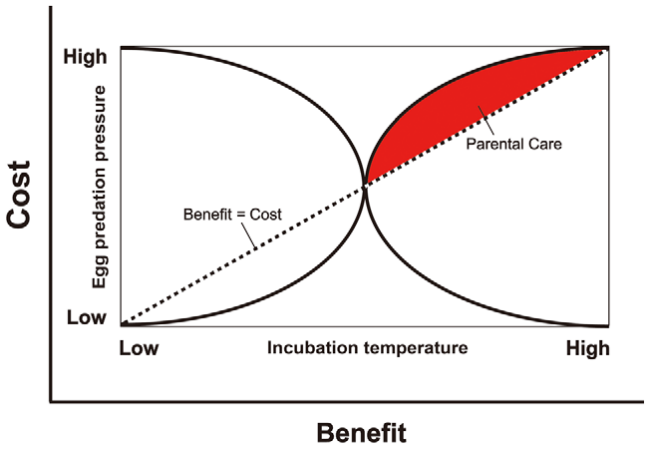
A hypothetical cost-benefit model describing the evolution of parental care in long-tailed skinks (*Eutropis longicaudata*), showing how parental care can evolve based on the relationship between incubation temperature (the benefit is increased hatching success, x-axis) and egg predation pressure from snakes (the cost of laying eggs in populations with varying snake population size, y-axis). Note that these relationships are hypothetical, and provide only one example of how these two variables could influence the evolution of maternal care (similar relationships can be developed between other variables of interest). Parental care has a high likelihood of evolving in instances where female lizards select nest sites that are relatively high (i.e., inside of a retaining wall as compared to in natural habitats) but in which there are large numbers of egg-eating snakes (and thus high risk of any eggs left unattended becoming depredated). This matches the situation on Orchid Island, Taiwan. Maternal care is unlikely to evolve when predation pressure is relatively low, which matches the situation on Green Island and mainland Taiwan.

Although the costs and benefits of parental care are many, we currently know little of the form of the relationships between parental investment and offspring fitness. It seems likely that this relationship could involve one or more threshold effects (e.g., see Fig. 1 for one putative relationship between selecting nest sites with appropriate incubation temperatures and predation pressure). However, there are many problems associated with measuring the costs of reproduction to the parents. Individual variation in phenotypic or genetic quality may obscure the effects of parental expenditure on subsequent fitness. Furthermore, experimental manipulation of parental expenditure is needed, even though it is difficult to measure reproductive costs and offspring benefits using either laboratory of field experiments [2]. Theoretical models of parental investment necessarily incorporate assumptions about the form of cost and benefit functions. Because it is impossible to deal with all of these functions adequately, few predictions concerning the form or extent of parental investment in practice have a basis grounded in ecology. For example, we do not currently know whether parents should generally invest more heavily in offspring of superior quality because they are more likely to survive, or inferior ones because parental expenditure will have a greater influence on their fitness. Because we know nothing about whether environmental factors affect the parents' decisions, those uncertainties lead us to be skeptical of predictions based only on theoretical principles. This may be a problem in our model as well, but our goal was to use biologically meaningful parameters to understand how these might relate to parental care evolution.

### Why the mainland Taiwan population has not evolved maternal care

There are two explanations for why the long-tailed skink population on mainland Taiwan has not evolved maternal care. First, on the mainland there is a substantial risk of predation to the parent while guarding the nest because of the diverse array of lizard predators there (e.g., at least 4 snake species; Table 1 [9]). The presence of a higher diversity of lizard predators could increase C_risk_. If the mother lizard is preyed upon, then the X value will increase simultaneously because the eggs are vulnerable to predators without maternal care. Secondly, experimental exclusion of vertebrate predators in the mainland population revealed that the benefits of care and desertion are almost equivalent in terms of hatching success (Table 1 [9]). Excluding vertebrate predators did not result in a significant increase in hatching success on mainland Taiwan or Green Island, as compared to a 63% increase for the Orchid Island population (Table 1 [9]). When there is no positive net benefit to maternal care, natural selection will favor nest desertion.

Our cost-benefit model predicts that animals will seek further benefits if the environment allows it, regardless of whether the species shows aggression towards egg predators. For example, higher temperatures influence egg incubation time and egg mortality of long-tailed skinks on Orchid Island [11]. Eggs incubated in the warmer concrete wall have higher hatching success (88%) than natural nests (67%), and hatch 7 days earlier [11]. In warmer environments, shorter incubation times could reduce the duration of maternal care (when the female remains at the nest for the entire duration of incubation) and allow the hatchlings to have longer activity seasons (and thus reach larger sizes) prior to winter. Thus, these concrete walls are likely to attract female lizards because of the benefits to incubating eggs, regardless of whether that female has the ability to deter predators or even provide maternal care at all. The Taiwanese and Green Island populations are consistent with this prediction in that they have not evolved maternal care, but do lay their eggs inside the concrete walls.

Predation is a strong selective force that influences the behavior, morphology and life history of prey species [17]–[18]. Studies of the effects of predation on prey responses are biased towards studies of life history and morphology. Nevertheless, exposure to predators has been shown to influence prey behavioral changes and evolution (e.g. [18]–[20]). The current cost-benefit model suggests that both individual predators and reptile egg predators are important to the evolution of maternal care in long-tailed skinks. Direct contact between prey and potential predators is unlikely to induce parental care in smaller species on the island, because they are unable to successfully deter egg-eating snakes. However, when predators are rare (or multiple predators co-occur) it may be difficult to evolve maternal care because the costs of providing care may not outweigh the benefits. For example, the Taiwanese population of skinks is exposed to many different predators, many of which eat lizards. Likewise, on Green Island there are very few predators at all, and consequently maternal care would accrue only minimal benefits (Table 1).

### The cost and benefit model in a hypothesis-testing framework

Existing hypotheses about the mechanisms behind the evolution of parental care [1] can be integrated into our findings, which results in eight hypotheses spanning: (1) attributes of the external environment, including nesting sites; and (2) reproductive attributes of the species, both of which must result in the parental action increasing fitness. We address each of these hypotheses in turn.

### Characteristics of the external environment

#### 1. Harsh and unpredictable environments

This hypothesis suggests that parental care will most often be found in environments in which resources for adults are limited at the time of egg guarding [21]. For example, if resources are scarce prior to or during the period of parental care, then the parents might increase the rate of food searching while guarding the nest, which could increase the risk of predation to the parent or offspring, which would reduce the benefits of providing maternal care [21]. However, if food resources are abundant at the time of nesting, an alternative hypothesis is that parents would not necessarily tradeoff egg guarding with food searching. This would lessen the time spent away from the nest and provide the female with more energy with which to protect the nest. Parental care might strongly benefit egg survivorship in these instances. In our current study, for instance, long-tailed skinks breed from February to September when the food resources are abundant [14], in contrast to the harsh and unpredictable environment hypothesis, but in support of the abundant food resource hypothesis. Although skinks occasionally leave the nest to forage in the forest near the concrete wall [9], these excursions may be infrequent enough or of sufficiently short duration that foraging does not increase the risk of predation to the mother or eggs. Addressing this hypothesis using more detailed behavioral observations will help clarify this.

#### 2. Selection for “risky” life history strategies

Our model predicts that parental care will evolve more commonly when reproductive output is low. This implies that short-lived species are more likely to evolve parental care than long-lived species. Short-lived species often have lower reproductive outputs than long-lived species, and thus could be more likely to pursue “risky” reproductive strategies than long-lived ones [1], [22]. Parental care is a “risky” behavior because it can render the parents vulnerable to predators. In lizards, parental care is more common in late-maturing or large species [22], [23]. Tinkle (1969) suggested that parental care generally should be found in long-lived iteroparous species, especially those with a short, annual breeding season. However, the long-tailed skink, an iteroparous species with a long breeding season and large body size relative to other sympatric lizards, does not support this prediction. This inconsistency might correlate with a lizard's ability to defend against predators. For example, *Sphenomorphus incognitus*, a lizard with a small body size, cannot defend its nest from *Oligodon formosanus* [24] because its head width (8.1–10.9 mm; [25]) is smaller than the chest diameter of *O. formosanus* (11.5–14.5 mm; [26]), but the head width of *Eutropis* females (13.3–18.3 mm; [14]) is larger than the chest diameter of the snake; thus they can defend nests from intruding snakes.

#### 3. Exposed vs hidden nest sites

Tinkle (1969) reviewed parental care of lizards and suggested that lizards laying their eggs in well-hidden nests might be less exposed to dangers than a female that does not accompany her eggs. Likewise, eggs incubating in exposed areas, rather than buried or hidden, may be easier for predators to locate. Hence, parental care should evolve more often in species that do not bury their eggs [1], [27]. Although long-tailed skinks bury their eggs beneath rocks in natural habitats, the eggs of females nesting inside the retaining wall remain exposed during incubation. Females nesting in populations expressing or not expressing maternal care both nest in these retaining walls, suggesting that egg exposure in and of itself does not always lead to maternal care.

#### 4. Suitability of habitat for clutch attendance

Parental care is predicted to be more common when adults occupy the same habitat in which eggs are laid. This is because the parents may be less susceptible to predation inside their own territory, which also provides vital resources. This hypothesis is difficult to test in lizards because the males are more likely to be territorial, although females are more likely to show parental care [1]. Long-tailed skinks do not support this prediction because females generally spend the period outside the nesting season in natural habitats, but move onto the retaining wall during the nesting season [10]. A related hypothesis concerns the abundance of nest-sites available. Parental care might evolve when nest sites are scarce relative to the number of nesting females, because the arrival of new females can destroy existing nests. This scenario has been described in sea turtles [28] and island populations of iguanine lizards [29]. However, at our study areas there are nearly 1,200 identical potential nest-sites available, but we have never observed more than 50 nesting females at any given time. Because the number of suitable nesting sites vastly outnumbers the number of breeding females, our data do not support this prediction.

#### 5. Ability of parents to defend eggs from predators

If a major benefit of parental care is to deter potential egg-predators, then parental care should evolve most often in species in which a parent is physically capable of deterring egg predators [1], [3], [30]. For example, parental care is common in the largest snakes (Pythoninae), and those with venom (Viperidae, >50% of species and Elapidae, 41% of species). Likewise, the few reports of parental care in the Colubridae (7.5%) are in species with relatively large body sizes (e.g., the genera *Elaphe, Farancia, Ptyas*) or belonging to the minority of venomous species within the family (e.g., *Psammophylax, Rhabdophis*; [1]). The ubiquity of parental care in the large and formidable crocodilians is consistent with this prediction. Likewise, due to their relatively large body size, long-tailed skinks appear to be the only lizard species on Orchid Island large enough to successfully deter egg-eating snakes [24].

### Reproductive attributes

#### 6. Parental care is more likely to evolve in species with low reproductive frequency

Parental care may be more likely to evolve in species that produce only a single clutch of large eggs per reproductive season, as opposed to species that produce small and frequent clutches. This is because the cost of caring for a single clutch of eggs is most likely smaller than the cost of caring for several clutches over a much longer duration. Data from several studies seemingly support this prediction [23], [31]. However, because multiple clutching is more common in tropical species than in temperate species, parental care should evolve more often in temperate rather than tropical species. This is not the case for reptiles, in which parental care is most common in tropical species (e.g., Iguaninae, Pythoninae, Alligatorinae, Crocodylinae [1]). The costs for long-tailed skinks to guard their eggs are minimal, and similar for females with both large and small clutches [13]. Some long-tailed skinks reproduce twice within a single breeding season, and guard both clutches, which does not support this prediction.

#### 7. Brief incubation periods

Parental care should evolve when incubation periods are short because females investing less time engaged in parental care will incur fewer costs. A comparison of the incubation periods of egg-attending vs non-attending species supports this hypothesis [21]. However, such interspecific comparisons could be confounded if large species that tend to have large eggs (which need longer incubation periods) are disproportionately represented in the non-attending species. Our field data support hypothesis; long-tailed skink eggs incubate more quickly on Orchid Island than on the mainland [13]. However, the incubation period may not be a major factor in the evolution of maternal care in this species because females in populations with and without maternal care nest in two habitat types: some females bury eggs beneath the soil (where they are not visible to predators) and some females lay eggs inside of a retaining wall (where they are visible to predators; [32]). Furthermore, the duration of maternal care does not always last the entire incubation period, but can vary due to the frequency of attempted egg predation by snakes [9]. Hence, this prediction may be overly simplistic.

### Parental action enhancing fitness

#### 8. Parental care is more likely to evolve when it increases egg hatching ratios

According to our model, the higher the hatching ratio (in terms of benefit, B_egg_) the greater the opportunity for parental care evolution. For example, long-tailed skink eggs laid inside concrete walls have higher hatching success than those in natural nests [11]. Exposing the eggs to higher incubation temperatures might play an important role in the evolution of parental care. This prediction may help explain why tropical species more commonly display parental care than temperate species [1]. Eggs in tropical environments are exposed to higher temperatures, and in many species this results in higher hatching success, which would bring the benefits in line with our model. Further, our model might predict that endotherms would show parental care more commonly than ectotherms, because most of the temperatures experienced by embryos are higher in ectotherms than in ectotherms. This could help explain why almost all birds and mammals show parental care.

### Conclusions

We know so little about the mechanisms leading to the evolution of parental care that it is difficult to predict how parents should invest in their offspring. Our general cost-benefit framework can be used to examine how a relatively large number of biologically-relevant variables simultaneously influence quantitative estimates of parental care. This approach revealed that the evolution of maternal care in long-tailed skinks is related to at least five different factors: the energetic investment in a clutch, the delay of future fecundity, the risk of predation to the parent, the ability to defend the eggs from predation, and the increase in hatching success provided by maternal care. The relative magnitudes of the cost and benefits of these variables are important in determining the evolution of parental care. Consequently, multiple pathways can lead to the evolution of parental care from a non-caring state, even in a single population of a widespread species.

## References

[1] Shine R (1988) *Parental care in reptiles*, Pages 275–330 *in* Gans C, Huey RB, eds. *Biology of the Reptilia Vol.16*. Alan R. Liss. New York.

[2] Clutton-Brock TH (1991) The evolution of parental care: Monographs in behavior and ecology. Princeton University Press.

[3] Greene HW, May PG, Hardy SDL, Sciturro JM, Farrell TM (2002) *Parental behavior by vipers*, Pages 179–205 *in* Schuett GW, Hoggren M, Douglas ME, Greene HW, eds. *Biology of the Vipers*. Eagle Mountain Publ. Eagle Mountain, UT.

[4] Reguera P, Gomendio M (1999) Predation costs associated with parental care in the golden egg bug *Phyllomorpha laciniata* (Heteroptera: Coreidae). Behav Ecol 10: 541–544.

[5] Winkler DW (1987) A general model for parental care. Am Nat 130: 526–543.

[6] Reynolds JD, Gross MR (1990) Costs and benefits of female mate choice: is there a lek paradox? Am Nat 136: 230–243.

[7] Rosenheim JA (1999) Characterizing the cost of oviposition in insects: a dynamic model. Evol Ecol 13: 141–165.

[8] Besnard A, Gimenez O, Lebreton JD (2002) A model for the evolution of creching in gulls. Evol Ecol 16: 489–503.

[9] Huang WS, Lin SM, Dubey S, Pike DA (In press) Predation drives interpopulation differences in parental care expression. J Anim Ecol *in press*. 10.1111/1365-2656.12015 23237108

[10] Huang WS (2006a) Parental care in the long-tailed skink, *Mabuya longicaudata* on a tropical Asian island. Anim Behav 72: 791–795.

[11] Huang WS, Pike DA (2011) Climate change impacts on fitness depend on nesting habitat in lizards. Funct Ecol 25: 1125–1136.

[12] Maynard-Smith J (1977) Parental investment: A prospective analysis. Anim Behav 25: 1–9.

[13] Huang WS (2007) Costs of egg caring in the skink, *Mabuya longicaudata* . Ecol Res 22: 659–664.

[14] Huang WS (2006b) Ecological characteristics of the skink, *Mabuya longicaudata*, on a tropical East Asian island. Copeia 2006: 293–300.

[15] Schmitz OJ, Cohon JL, Rothley KD, Beckerman AP (1998) Reconciling variability and optimal behaviour using multiple criteria in optimization models. Evol Ecol 12: 73–94.

[16] Stearns SC (1992) The evolution of life histories. Oxford University Press, Oxford UK.

[17] Lima SL, Dill LM (1990) Behavioral decisions made under the risk of predation: a review and prospectus. Canad J Zool 68: 619–640.

[18] Chivers DP, Kiesecker JM, Marco A, DeVito J, Anderson MT, et al (2001) Predator-induced life history changes in amphibians: egg predation induces hatching. Oikos 92: 135–142.

[19] Crowl TA, Covich AP (1990) Predator-induced life history shifts in a freshwater snail. Science 247: 949–951. 17776452 10.1126/science.247.4945.949

[20] Ball SL, Baker RL (1996) Predator-induced life history changes: antipredator behavior costs or facultative life history shifts. Ecology 77: 1116–1124.

[21] Tinkle DW, Gibbons JW (1977) The distribution and evolution of viviparity in reptiles. Misc. Pub. Museum Zool. Univ. Mich. 154: 1–54.

[22] Williams GC (1966) Adaptation and natural selection. Princeton University Press, Princeton, NJ.

[23] Tinkle DW (1969) The concept of reproductive effort and its relation to the evolution of life histories of lizards. Am Nat 103: 501–516.

[24] Tseng HY, Huang WS (2012) *Sphenomorphus incognitus* (Brown forest skink). Parental care. Herpetol Rev 43: 141.

[25] Huang WS (2010) Foraging behaviors of two sympatric ant species in response to lizard eggs. Zoology 113: 85–90. 20199855 10.1016/j.zool.2009.06.003

[26] Huang WS (2004) Reptile ecology and the evolution of parental care on a tropical Asian island. Ph. D thesis, Cornell University, NY.

[27] Noble GK, Mason ER (1933) Experiments on the brooding habits of the lizards *Eumeces* and *Ophisaurus* . Am Mus Novit 619: 1–29.

[28] Girondot M, Tucker AD, Rivalan P, Godfrey MH, Chevalier J (2002) Density-dependent nest destruction and population fluctuations of Guianan leatherback turtles. Anim Conserv 5: 75–84.

[29] Rand AS (1968) A nesting aggregation of iguanas. Copeia 1968: 552–561.

[30] Fraipont MD, Clobert J, Barbault R (1996) The evolution of oviparity with egg guarding and viviparity in lizards and snakes: a phylogenetic analysis. Evolution 50: 391–400. 28568867 10.1111/j.1558-5646.1996.tb04501.x

[31] Bull JJ, Shine R (1979) Iteroparous animals that skip opportunities for reproduction. Am Nat 114: 296–316.

[32] Huang WS (1994) Report on egg clutch size of long-tailed skink, *Mabuya longicaudata* from Taiwan. J Taiw Muse 47: 45–47.

[33] Huang WS, Greene HW, Chang TJ, Shine R (2011) Territorial behaviour in Taiwanese kukrisnakes (*Oligodon formosanus*). Proc Natl Acad Sci 108: 7455–7459. 21502515 10.1073/pnas.1101804108 PMC3088593

